# Regulation of *Arabidopsis thaliana* seed dormancy and germination by 12-oxo-phytodienoic acid

**DOI:** 10.1093/jxb/erw028

**Published:** 2016-02-11

**Authors:** Anuja Dave, Fabián E. Vaistij, Alison D. Gilday, Steven D. Penfield, Ian A. Graham

**Affiliations:** ^1^Centre for Novel Agricultural Products, Department of Biology, University of York, York YO10 5DD, United Kingdom; ^2^Department of Crop Genetics, John Innes Centre, Norwich NR4 7UH, United Kingdom

**Keywords:** Abscisic acid, dormancy, germination, MFT, OPDA, RGL2.

## Abstract

We previously demonstrated that the oxylipin 12-oxo-phytodienoic acid (OPDA) acts along with abscisic acid to regulate seed germination in *Arabidopsis thaliana*, but the mechanistic details of this synergistic interaction remain to be elucidated. Here, we show that OPDA acts through the germination inhibition effects of abscisic acid, the abscisic acid-sensing ABI5 protein, and the gibberellin-sensing RGL2 DELLA protein. We further demonstrate that OPDA also acts through another dormancy-promoting factor, MOTHER-OF-FT-AND-TFL1 (MFT). Both abscisic acid and MFT positively feed back into the OPDA pathway by promoting its accumulation. These results confirm the central role of OPDA in regulating seed dormancy and germination in *A. thaliana* and underline the complexity of interactions between OPDA and other dormancy-promoting factors such as abscisic acid, RGL2, and MFT.

## Highlight

We demonstrate that 12-oxo-phytodienoic acid acts together with ABA in a complex regulatory network to promote seed dormancy and repress germination in Arabidopsis.

## Introduction

Seed dormancy prevents germination until conditions are favourable for plant growth. In *Arabidopsis thaliana* (Arabidopsis), dormancy can be released by exposing imbibed seeds to low temperatures (i.e. stratification) or by an extended period of dry seed storage (i.e. after-ripening). Plant hormones are chemically diverse compounds, which occur in plants at low concentrations and regulate plant growth, development, and responses to external stimuli (Davies, 2010). Among these, the two hormones that are of the utmost importance in seed dormancy and germination regulation are abscisic acid (ABA) and gibberelic acid (GA). ABA plays a key role in induction of primary dormancy during Arabidopsis seed development and acts as a germination repressor, while GA promotes germination and the alleviation of dormancy (Finkelstein *et al.*, 2008; Graeber *et al.*, 2012; Holdsworth *et al.*, 2008). Both biosynthesis and catabolism determine the final amount of ABA and GA in the seed, and the interactions between these two antagonistic hormones, at the metabolism and signalling levels, are crucial in the regulation of germination.

The transcription factors ABI3, ABI4, and ABI5 are important components of the ABA signal transduction pathway involved in germination regulation (Clerkx *et al.*, 2003; Finkelstein, 1994; Finkelstein and Lynch, 2000; Finkelstein *et al.*, 1998; Lopez-Molina and Chua, 2000; Lopez-Molina *et al.*, 2002; Xu *et al.*, 2014). ABI5 is a basic leucine-zipper (bZIP) transcription factor (Finkelstein and Lynch, 2000; Lopez-Molina and Chua, 2000); in Arabidopsis seeds it is expressed in the embryo and micropylar endosperm (Penfield *et al.*, 2006a). ABA causes an increase in *ABI5* transcript levels and protein abundance, and also activates ABI5 via phosphorylation (Lopez-Molina *et al.*, 2001; Piskurewicz *et al.*, 2008). Integral to GA signal transduction are the growth-repressing DELLA proteins. GA binds to the receptor GA INSENSITIVE DWARF1 (GID1) and enhances GID1-DELLA interaction, leading to DELLA degradation by the ubiquitin-proteasome pathway (Murase *et al.*, 2008; Sun, 2010). DELLA proteins repress germination, and of the five *DELLA* genes in Arabidopsis, *RGA-LIKE2* (*RGL2*) is the major DELLA involved in seed germination (Cao *et al.*, 2005; Lee *et al.*, 2002; Penfield *et al.*, 2006b; Piskurewicz and Lopez-Molina, 2009; Piskurewicz *et al.*, 2008; Tyler *et al.*, 2004). RGL2 accumulation can also lead to an increase in ABA levels via regulation of the RING-zinc finger protein-encoding gene *XERICO*, which has been shown to promote accumulation of ABA in an as yet unknown manner (Ko *et al.*, 2006; Piskurewicz *et al.*, 2008; Zentella *et al.*, 2007).

FLOWERING-LOCUS-T (FT), TERMINAL-FLOWER1 (TFL1), and MOTHER-OF-FT-AND-TFL1 (MFT) belong to the phosphatidyl ethanolamine-binding protein family of proteins in Arabidopsis. While FT and TFL1 are involved in regulation of flowering time control, MFT has been shown to be a regulator of germination and dormancy in different species (Li *et al.*, 2014; Nakamura *et al.*, 2011; Vaistij *et al.*, 2013; Xi and Yu, 2010; Xi *et al.*, 2010). Data reported by Vaistij *et al.* (2013) and Nakamura *et al.* (2011) indicate that MFT promotes dormancy, and this is consistent with the fact that *MFT* expression is promoted by RGL2 and ABI5 in Arabidopsis (Xi *et al.*, 2010). However, MFT has also been found to negatively regulate ABA signalling (Xi *et al.*, 2010).

Jasmonic acid (JA) and its precursor 12-oxo-phytodienoic acid (OPDA) are oxylipins derived from α-linolenic acid (Wasternack and Hause, 2013). JA regulates biotic and abiotic stress responses, as well as plant growth and developmental processes (Balbi and Devoto, 2008; Santino *et al.*, 2013; Wasternack, 2014; Wasternack *et al.*, 2013). OPDA has also been shown to possess distinct, JA-independent, signalling properties in different species, and its involvement has been reported in biotic and abiotic stress responses, embryo development, and seed germination (Bosch *et al.*, 2014; Dave and Graham, 2012; Dave *et al.*, 2011; Goetz *et al.*, 2012; Guo *et al.*, 2014; Park *et al.*, 2013; Ribot *et al.*, 2008; Satoh *et al.*, 2014; Savchenko and Dehesh, 2014; Savchenko *et al.*, 2014; Taki *et al.*, 2005). A role for OPDA in repressing germination in Arabidopsis was proposed based on analysis of *comatose* (*cts*) seeds disrupted in the activity of the ATP binding cassette (ABC) transporter CTS, also known as PXA1 or PED3 (Footitt *et al.*, 2002; Hayashi *et al.*, 2002; Russell *et al.*, 2000; Zolman *et al.*, 2001). CTS is involved in the transport of OPDA into the peroxisome, where JA biosynthesis occurs. *cts*-2/*pxa1*-1 seeds accumulate high levels of OPDA, leading to the low germination rates observed for this mutant (Dave *et al.*, 2011). Moreover, exogenous OPDA leads to an increase in ABI5 protein abundance and inhibition of the germination of wild-type (WT) seeds (Dave *et al.*, 2011).

Here, we show that ABA, ABI5, RGL2, and MFT are key components of the OPDA pathway that represses germination. We also show that there is a complex network of feedback interactions by which these components downstream of OPDA affect OPDA levels in seeds.

## Materials and methods

### Plant and growth conditions

*Arabidopsis thaliana* WT and mutant plants used were in the Columbia-0 (Col), Landsberg *erecta* (L*er*) and Wassilewskija (Ws) ecotypes as stated. The mutants used in this study have been described previously: *pxa1*-1 (Zolman *et al.*, 2001), *cts*-2 (Footitt *et al.*, 2002), *aos* (Park *et al.*, 2002), *opr3*-1 (Stintzi and Browse, 2000), *rgl2*-1 (Lee *et al.*, 2002), *aba1*-1 (Koornneef *et al.*, 1982), *mft*-2 (Xi *et al.*, 2010), and *ga1*-3 (Sun and Kamiya, 1994). Plants were grown in controlled environment growth cabinets at 22 °C with 16h white light at 80–100 µmol m^−2^ s^−1^ light intensity. Seeds were harvested from brown siliques that had begun to dehisce, and were size-sieved using a 250 µm mesh sieve. Experiments performed with freshly harvested seeds were conducted within 24h of harvest. For the experiments with after-ripened seeds, freshly harvested seeds were dry stored for 6–8 weeks. For stratification, seeds were imbibed on water agar plates and stratified in the dark at 4 °C for 3 days before being placed in growth cabinets.

### Seed germination assays

Germination assays were performed on 0.9% (w/v) water agar plates, with 50–100 sterilized seeds, and plates were placed in a controlled environment growth cabinet with continuous light (150 µmol m^−2^ s^−1^) at 20 °C. In experiments where germination assays were conducted with OPDA (Larodan), ABA (Sigma), Norflurazon (Sigma), and Paclobutrazol (Sigma), the appropriate amounts of these compounds were included in the water agar medium. Germination was scored as radicle emergence after 7 days of incubation.

### Hormone and oxylipin analysis

ABA, GA, OPDA, and JA-isoleucine (JA-Ile) conjugate were extracted and quantified from dry seed (80–100mg dry weight) and imbibed seed (300–400mg fresh weight) according to the protocol described for GA and ABA quantification (Dave *et al.*, 2011), with d_2_-GA4, d_6_-ABA and Prostaglandin A1 included as internal standards for GA_4_, ABA, and OPDA/JA-Ile, respectively.

### Gene expression analysis

RNA extractions from developing seeds and imbibed seeds, cDNA synthesis, and quantitative PCR analysis were performed according to methods described previously (Penfield *et al.*, 2005; Vaistij *et al.*, 2013). cDNA amplifications were normalized to *ACTIN2* and relative gene expression was calculated as a ratio between the value of each sample and their respective controls. The primers used are described in Supplementary Table 1 at *JXB* online.

### Statistical analysis

In all Figures the data represent the mean of three biological replicates±standard deviation. Differences between controls and samples stated in the text are statistically significant as determined by Student’s *t*-test (*P*≤0.05).

## Results and discussion

### Seed dormancy is affected in OPDA biosynthetic and catabolic mutants

**Fig. 1.**
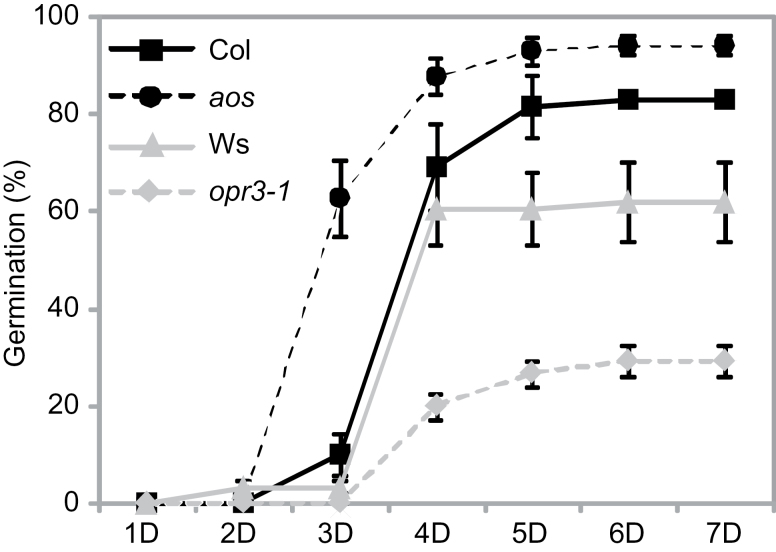
Seeds affected in OPDA metabolism have altered dormancy. Germination profiles of WT Col and Ws, *aos* (in Col background), and *opr3*-1 (in Ws background) freshly harvested, unstratified seeds from 1 to 7 days after imbibition (1D–7D) on water agar plates under constant light. In this and all other Figures, error bars represent the standard deviation of three independent measurements.

We demonstrated previously that Arabidopsis *pxa1*-1/*cts*-2 mutant seeds are highly dormant due to an over-accumulation of OPDA (Dave *et al.*, 2011). We also showed that seeds of the *aos* mutant (disrupted in an early step in the oxylipin pathway prior to OPDA; Park *et al.*, 2002) and of the *opr3*-1 mutant (unable to convert OPDA into JA; Stintzi and Browse, 2000) have lower and higher levels of OPDA, respectively, than WT seeds (Dave *et al.*, 2011). Based on these observations, we hypothesized that seeds of these mutants should also have altered dormancy levels correlating with their OPDA levels in seeds. To test this hypothesis, germination levels of freshly harvested seeds were determined. We found that *aos* seeds, which are deficient in OPDA accumulation, indeed germinated faster and at a higher frequency than WT (Col) seeds (Fig. 1). In contrast, *opr3*-1 seeds, which contain more OPDA, germinated at a lower frequency than their respective WT (Ws) seeds (Fig. 1). These observations further confirm that levels of dormancy positively correlate with OPDA accumulation in freshly harvested seeds.

### OPDA acts through the ABA pathway

**Fig. 2.**
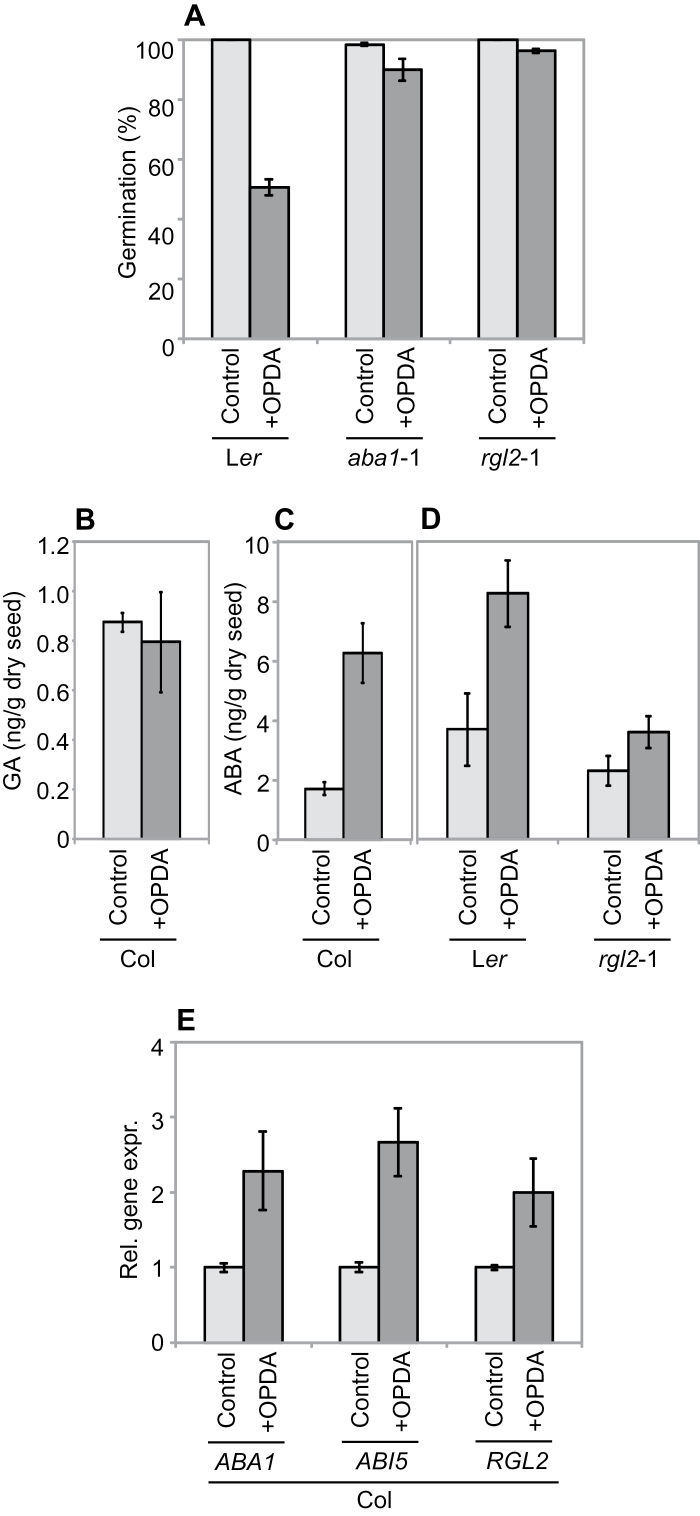
The ABA biosynthetic pathway is required for OPDA-driven inhibition of germination. (A) Germination profiles [7 days after imbibition (DAI)] of after-ripened WT (L*er*), *aba1*-1 and *rgl2*-1 seeds on water agar (Control) plates and water agar supplemented with 10 µM OPDA (+OPDA). (B–D) GA (GA4) and ABA levels in after-ripened WT (Col and L*er*) and *rgl2*-1 seeds on Control or +OPDA plates measured 2 DAI. (E) Relative gene expression of *ABA1*, *ABI5*, and *RGL2* in after-ripened seeds.

We showed in our previous work that exogenously applied OPDA and ABA synergistically inhibit germination of after-ripened seeds (Dave *et al.*, 2011). Here, we extended our analyses by performing germination assays using seeds of the ABA-biosynthesis mutant *aba1*-1 and the GA-signalling mutant *rgl2*-1. As shown for Col and Ws (Dave *et al.*, 2011), germination of after-ripened L*er* seeds was inhibited by exogenously applied OPDA (Fig. 2A). However, OPDA was almost ineffective when germination assays were performed with *aba1*-1 and *rgl2*-1 seeds (Fig. 2A). The ABA-dependent effect of OPDA was also observed when WT (Col) seeds were treated jointly with OPDA and the indirect ABA biosynthesis inhibitor Norflurazon, to mimic the *aba1*-1 mutation (Supplementary Fig. S1). We also measured GA and ABA accumulation in OPDA-treated seeds and found that while GA levels were unchanged (Fig. 2B), ABA accumulation was increased in both Col and L*er* WT seeds (Fig. 2C, D). However, the ABA increase was strongly reduced in *rgl2*-1 mutant seeds (Fig. 2D). This is in accordance with previous observations showing that RGL2 indirectly promotes ABA biosynthesis (Lee *et al.*, 2010). Taken together, these observations demonstrate that exogenously applied OPDA triggers ABA accumulation. 

We then investigated whether elevated levels of endogenous OPDA in seeds also correlated with increased amounts of ABA. To do this, we quantified ABA in OPDA over-accumulating *cts*-2 seeds (Dave *et al.*, 2011) and found that, as with OPDA-treated seeds, their ABA levels were increased (Supplementary Fig. S2).

In water-stressed Arabidopsis roots, induction of the JA-Ile conjugate, but not OPDA, is required for ABA accumulation in an *NCED3*-dependent manner (De Ollas *et al.*, 2015). *NCED3* is a key gene regulating ABA biosynthesis under stress conditions (Nambara and Marion-Poll, 2005). One possible explanation for the germination-inhibiting effect of OPDA is that OPR3 converts OPDA into JA/JA-Ile, which in turn promotes ABA biosynthesis to inhibit germination. However, *opr3*-1 seeds, which over-accumulate OPDA but not JA/JA-Ile (Dave *et al.*, 2011), are more dormant than WT seeds (Fig. 1). Additionally, exogenously applied OPDA represses germination of *opr3*-1 seeds as well as WT control seeds (Supplementary Fig. S3). Hence, in the germination inhibition response, it is OPDA rather than JA/JA-Ile that leads to the increase in ABA to inhibit germination. Thus, from our own work and that of others, it seems that both JA/JA-Ile and OPDA are able to affect ABA biosynthesis, but which compound does so depends on the tissue, stress stimuli, and developmental stage. Drought also induces OPDA and ABA accumulation, and these two compounds cooperatively regulate stomatal aperture in a JA-independent manner; however, in this case the response initiated by OPDA is unlikely to be via its effect on ABA biosynthesis (Savchenko *et al.*, 2014). Thus, a mode of action for OPDA whereby it acts along with ABA, but not through a direct effect on ABA biosynthesis, is also possible.

To gain more insight into the mechanism of OPDA-driven ABA accumulation in seeds, we analysed the expression of *ABA1* and *RGL2*. We found that exogenously applied OPDA promotes the expression of both genes (Fig. 2E). Thus, OPDA has the ability to regulate the expression of genes involved in ABA production, but it remains unknown how OPDA operates to achieve this. Work from other laboratories has shown that expression of the ABA-signalling gene *ABI5* is up-regulated by endogenous and exogenous ABA (Lopez-Molina *et al.*, 2001; Okamoto *et al.*, 2010; Piskurewicz *et al.*, 2008). This prompted us to assess whether exogenously applied OPDA has a similar effect on *ABI5* expression, and we found that it does (Fig. 2E). Hence, OPDA not only triggers ABA accumulation but can also increase ABA sensitivity. Furthermore, the importance of ABI5 in the OPDA-imposed dormancy pathway has been demonstrated *in planta* by studying *ped3* mutant seeds, which are deficient in the same ABC transporter as *cts*-2/*pxa1*-1. The *ped3* seeds are highly dormant, have increased *ABI5* expression, and are rescued by the *abi5* mutation (Kanai *et al.*, 2010). However, the regulation of ABI5 by OPDA does not occur exclusively at the level of gene expression, since OPDA also increases ABI5 protein stability (Dave *et al.*, 2011). Taken together, these observations show that OPDA promotes ABA biosynthesis by increasing *ABA1* and *RGL2* expression, and ABA sensitivity by increasing *ABI5* gene expression and ABI5 protein stability.

### ABA regulates OPDA accumulation

**Fig. 3.**
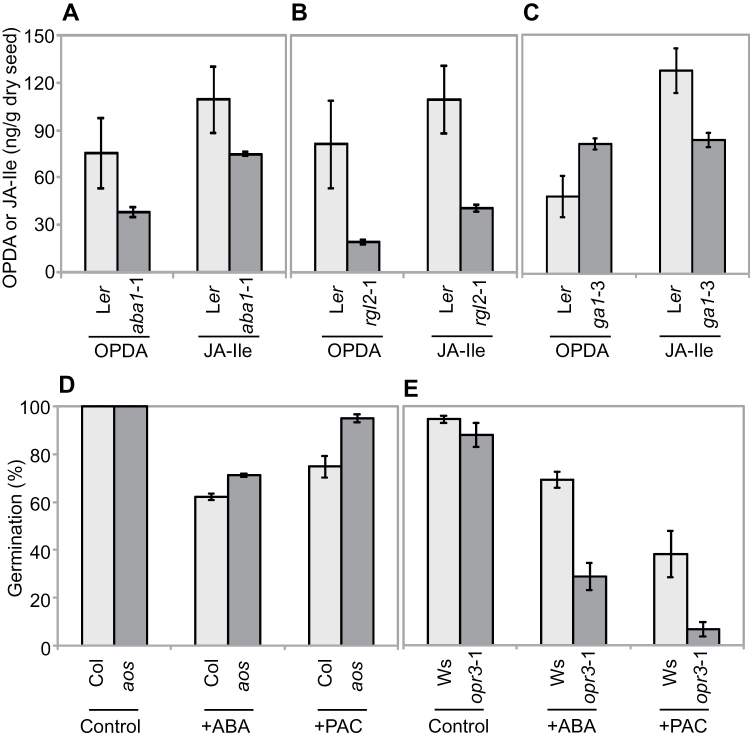
ABA affects the oxylipin pathway. (A–C) OPDA and JA-Ile levels in WT (L*er*), *aba1*-1, *rgl2*-1, and *ga1*-3 freshly harvested dry seeds. (D–E) Germination profiles (7 DAI) of WT (Col), *aos*, and *opr3*-1 freshly harvested, stratified (3 days in the dark at 4 °C), seeds on water agar (Control) plates and water agar supplemented with 2 µM ABA (+ABA) or 1 µM Paclobutrazol (+PAC).

It has been reported that Arabidopsis seedlings infected with the fungus *Phytium irregulare* accumulate JA and OPDA in an ABA-biosynthesis-dependent manner (Adie *et al.*, 2007). This led us to investigate a possible feedback of ABA and GA into oxylipin biosynthesis in seeds. Thus, OPDA and JA-Ile levels were measured in freshly harvested dry seeds of ABA/GA metabolism and signalling mutants. We found that *aba1*-1 and *rgl2*-1 seeds accumulate less OPDA and JA-Ile than WT (L*er*) controls (Fig. 3A, B). However, GA-deficient *ga1*-3 mutant seeds, which also accumulated lower levels of JA-Ile, contained a small but significant increase in OPDA levels (Fig. 3C). We also assessed whether the germination-inhibiting effects of ABA and Paclobutrazol (a GA-biosynthesis inhibitor) were dependent on OPDA by analysing *aos* and *opr3*-1 mutants. We found a small but significant reduction in the effect of ABA and Paclobutrazol on after-ripened *aos* mutant seeds, which lack OPDA (Fig. 3D). Consistent with this result, *opr3*-1 seeds, which accumulate OPDA, were found to be hypersensitive to ABA and Paclobutrazol (Fig. 3E).

Cross-talk between oxylipin (specifically JA) signalling and GA signalling has been reported previously in non-seed systems (Hou *et al.*, 2010; Navarro *et al.*, 2008; Qi *et al.*, 2014; Wild *et al.*, 2012; Yang *et al.*, 2012), but interaction between OPDA and GA at the level of biosynthesis has not been shown before. It is possible that the GA and OPDA interactions are indirect via ABA, since GA triggers degradation of RGL2, which in turn indirectly promotes accumulation of ABA and oxylipins (Figs 2E and 3B). Taken together, these observations demonstrate that the germination-inhibitory effects of ABA and Paclobutrazol are (at least partially) augmented by OPDA, and that there is a feedback from ABA and GA to regulate OPDA accumulation.

### OPDA and MFT interact reciprocally to inhibit germination

**Fig. 4.**
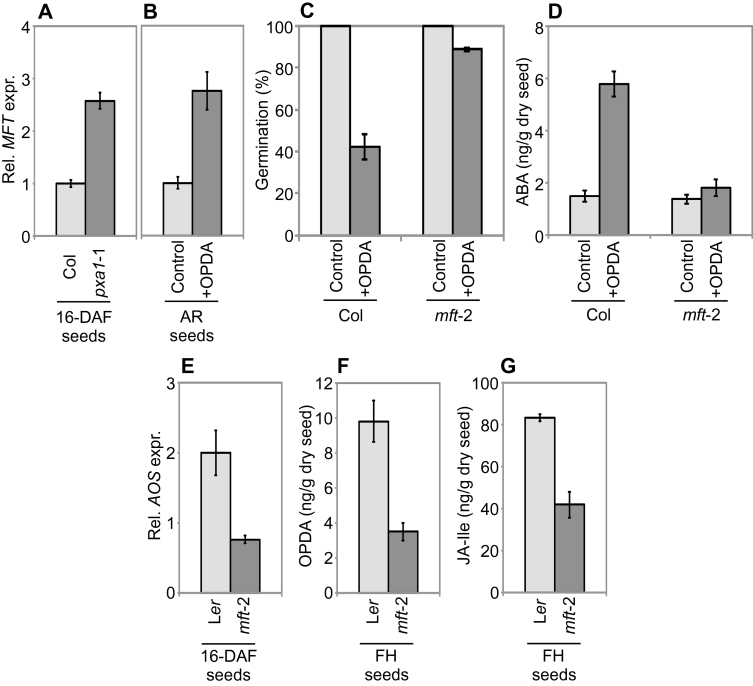
Interactions between OPDA and MFT. (A, B) Relative *MFT* expression in WT (Col) and *pxa1*-1 developing seeds 16 days after flowering (16-DAF) (A), and in after-ripened (AR) WT (Col) seeds on water agar (Control) plates and water agar supplemented with 10 µM of OPDA (+OPDA) (panel B). (C) Germination profiles (7 DAI) of WT (Col) and *mft*-2 after-ripened seeds on Control and +OPDA plates. (D) ABA levels in WT (Col) and *mft*-2 after-ripened seeds imbibed on Control and +OPDA plates measured 2 DAI. (E) Relative *AOS* expression in 16-DAF WT (Col) and *mft*-2 seeds. (F, G) OPDA and JA-Ile levels in WT (L*er*) and *mft*-2 freshly harvested (FH) dry seeds.

We reported previously that MFT plays a major role in promoting dormancy in Arabidopsis seeds (Vaistij *et al.*, 2013). This prompted us to assess whether the OPDA and MFT pathways are linked. Analysis of a transcriptomic dataset derived from *pxa1*-1 developing seeds (Dave *et al.*, 2011) revealed that *MFT* expression is higher in the mutant compared with WT. This observation was validated in independent samples of developing *pxa1*-1 seeds (Fig. 4A). Furthermore, MFT expression was also found to be elevated in OPDA-treated after-ripened WT seeds (Fig.4B). Thus, high endogenous or exogenous OPDA levels induce expression of the dormancy-promoting *MFT* gene. Interestingly, we also found that *mft*-2 after-ripened seeds are almost insensitive to OPDA (Fig. 4C), which shows that MFT is required for OPDA-induced inhibition of germination. In order to gain more insight into the interactions between OPDA and MFT, we assessed ABA levels in OPDA-treated *mft*-2 after-ripened seeds, and found that they were lower than the levels observed in WT (Col) controls (Fig. 4D). This suggests that in order to trigger ABA biosynthesis, in addition to RGL2 (Fig. 2D), OPDA also requires MFT. We also investigated possible MFT-to-OPDA feedback interactions. To do this, we assessed *AOS* expression and found that it is down-regulated in *mft*-2 developing seeds (Fig. 4E). Whether MFT regulates *AOS* expression directly or indirectly remains to be determined but, consistent with *AOS* expression levels, both OPDA and JA-Ile levels were found to be lower in *mft*-2 freshly harvested dry seeds than levels in WT seeds (Fig. 4F, G).

**Fig. 5.**
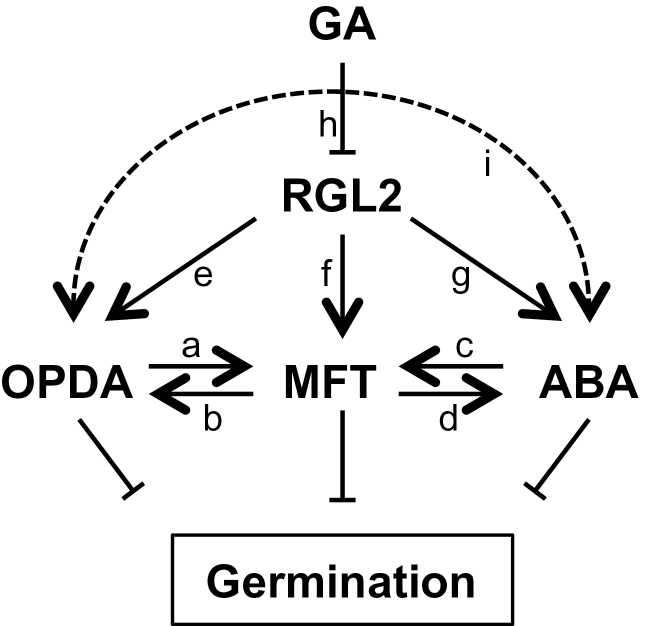
Model of interactions between the germination repressors OPDA, MFT, and ABA. OPDA promotes *MFT* gene expression (a; Fig. 4A, 4B) and MFT protein is required for OPDA accumulation (b; Fig. 4F). ABA also promotes *MFT* expression (c; Xi *et al.*, 2010) and MFT is required for OPDA-driven ABA accumulation (d; Fig. 4D). RGL2 is necessary for OPDA accumulation (e; Fig. 3B), *MFT* expression (f; Xi *et al.*, 2010), and OPDA-driven ABA accumulation (g; Fig. 2D). GA triggers RGL2 protein degradation (h; Murase *et al.*, 2008). The reciprocal positive feedback between OPDA and ABA (Figs 2C and 3A) may happen through MFT (a and d; c and b) or another indirect pathway (i). OPDA and MFT may *per se* have ABA-independent germination inhibitory effects. For simplicity, the involvement of ABI5 has not been depicted in this model.

Our results indicate that at least one role of the phosphatidyl ethanolamine-binding protein MFT and the DELLA protein RGL2 in the OPDA response is to elevate ABA levels. DELLA proteins are known to promote ABA accumulation by inducing *XERICO* expression, and XERICO protein promotes ABA accumulation by an unknown mechanism (Ko *et al.*, 2006; Piskurewicz *et al.*, 2008; Zentella *et al.*, 2007). MFT is also capable of influencing ABA biosynthesis by promoting *ABA1* expression (Li *et al.*, 2014). Previous work has shown that MFT also influences ABA sensing, since it negatively regulates *ABI5* and, in turn, *MFT* gene expression is promoted by ABI5 and RGL2 (Xi *et al.*, 2010). Our data demonstrate that in terms of germination, OPDA acts through MFT to promote both biosynthesis of and sensitivity towards ABA. Furthermore, RGL2 and MFT positively feed back on this regulation by promoting OPDA accumulation. However, we cannot exclude the possibility that OPDA and ABA also interact independently of MFT, or that OPDA directly inhibits germination independently of both ABA and MFT (Fig. 5).

In conclusion, cross-talk between different hormone pathways is known to occur in order to regulate physiological processes at various developmental stages and in response to external stimuli. In this work we have confirmed the role of OPDA as a potent dormancy-promoting/germination-inhibiting factor in Arabidopsis. We established that it acts in conjunction (but possibly not exclusively) with ABA, ABI5, RGL2, and MFT via a complex network of positive and negative feedback interactions. Further research is required to understand how OPDA is regulated by external stimuli and what other physiological processes can be influenced by OPDA in Arabidopsis.

## Supplementary data

Figure S1. OPDA requires ABA to inhibit germination.

Figure S2. OPDA and ABA levels are increased in *cts*-2 seeds.

Figure S3 *. opr3*-1 seeds are sensitive to OPDA.

Table S1. Sequence of primers used for gene expression analyses.

Supplementary Data
