# Supplementary material for: Regulation of Arabidopsis thaliana seed dormancy and germination by 12-oxo-phytodienoic acid
Source: J Exp Bot. 2016 Feb 11;67(8):2277–84. doi: 10.1093/jxb/erw028 (PMC4809285; doi:10.1093/jxb/erw028)
Supplement: Supplementary Data [file supp_67_8_2277__index.html]

Regulation of Arabidopsis thaliana seed dormancy and germination by 12-oxo-phytodienoic acid — Regulation of Arabidopsis thaliana seed dormancy and germination by 12-oxo-phytodienoic acid — Supplementary Data 

# Regulation of *Arabidopsis thaliana* seed dormancy and germination by 12-oxo-phytodienoic acid

## Supplementary Data

Data files

- Supplementary\_Figure\_S1\_S3\_Table\_S1.pdf - Supplementary Data
