# Supplementary material for: Regulation of Arabidopsis thaliana seed dormancy and germination by 12-oxo-phytodienoic acid
Source: J Exp Bot. 2016 Feb 11;67(8):2277–84. doi: 10.1093/jxb/erw028 (PMC4809285; doi:10.1093/jxb/erw028)
Supplement: Supplementary Data [file supp_erw028_Supplementary_Figure_S1_S3_Table_S1.pdf]

Regulation of *Arabidopsis thaliana* seed dormancy and germination by 12-oxo-phytodienoic acid.

Anuja Dave, Fabián E. Vaistij, Alison D. Gilday, Steven D. Penfield and Ian A. Graham

Supplementary Data

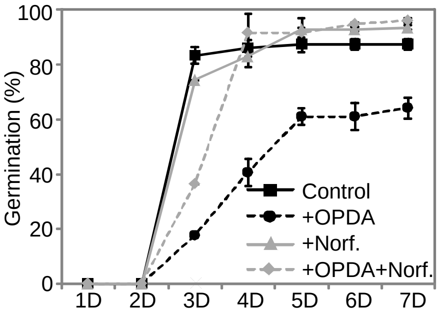

Supplementary Figure S1. OPDA requires ABA to inhibit germination: Germination assays of wild-type (Col) after-ripened seeds from 1 to 7 days after imbibition (1D–7D) on water agar (Control) plates and water agar supplemented with 10 µM OPDA (+OPDA), 5 µM Norflurazon (+Norf.), or both (+OPDA+Norf.).

Supplemental Figure S2

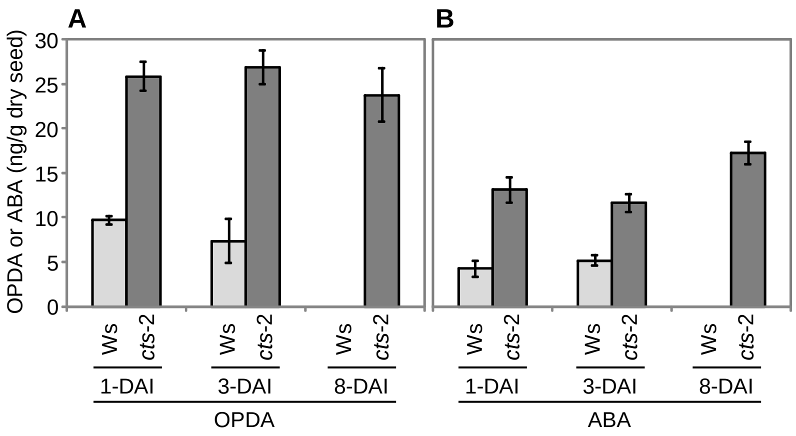

Supplementary Figure S2. OPDA and ABA levels are increased in *cts*-2 seeds: (A) OPDA and (B) ABA levels in wild-type (Ws) and *cts*-2 mutant seeds measured 1, 3 and 8 days after imbibition (DAI) on water agar plates.

Supplemental Figure S3

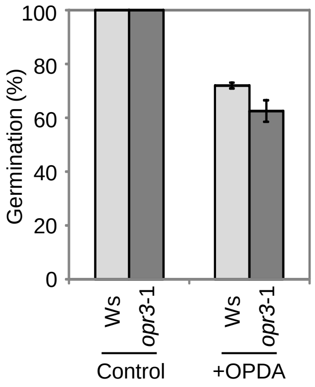

Supplementary Figure S3. *opr3*-1 seeds are sensitive to OPDA: Germination profiles of after-ripened wild-type (Ws) and *opr3*-1 seeds on water agar (Control) plates and water agar supplemented with 10 µM OPDA (+OPDA).

Supplementary Table S1

ACT-For	TGAGAGATTCAGATGCCCAGA
ACT-Rev	TGGATTCCAGCAGCTTCCAT
ABA1-For	ATGGCTTGCAATTATGGCTTC
ABA1-Rev	TCATCGGCTTTGTCAGTGAG
ABI5-For	CAATAAGAGAGGGATAGCGAACGAG
ABI5-Rev	CGTCCATTGCTGTCTCCTCCA
RGL2-For	CCGACCCGAATCTGAAACCTTAGTG
RGL2-Rev	AAGCGCTTCGTTGAACCTATCGAG
MFT-For	ATCACTAACGGCTGCGAGAT
MFT-Rev	CGGGAATATCCACGACAATC
AOS-For	AAGTCAAAGCCGGTGAAAT
AOS-Rev	CTTACCGGCGCATTGTTTAT

Supplementary Table S1. Sequence of primers used for gene expression analyses.
